# Phage Diversity in the Human Gut Microbiome: a Taxonomist’s Perspective

**DOI:** 10.1128/mSystems.00799-21

**Published:** 2021-08-17

**Authors:** Evelien M. Adriaenssens

**Affiliations:** a Quadram Institute Bioscience, Norwich, United Kingdom

**Keywords:** human gut virome, microbiome, phage taxonomy, phageome, virome

## Abstract

Bacteriophages (phages) have been known for over a century, but only in the last 2 decades have we really come to appreciate how abundant and diverse they are. With that realization, research groups across the globe have shown the importance of phage-based processes in a myriad of environments, including the global oceans and soils, and as part of the human microbiome. Through advances in sequencing technology, genomics, and bioinformatics, we know that the morphological diversity of bacteriophages originally used for taxonomy is eclipsed by their genomic diversity. Because we currently do not have a complete taxonomic framework or naming scheme to describe this diversity, crucial information from virome and microbiome studies is being lost. In this commentary, I will discuss recent advances in taxonomy and its importance for studies of the microbiome with examples of the human gut phageome and make recommendations for future analyses.

## COMMENTARY

## RECENT CHANGES IN PHAGE TAXONOMY AND THEIR IMPLICATIONS FOR MICROBIOME ANALYSES

**FIG 1:**
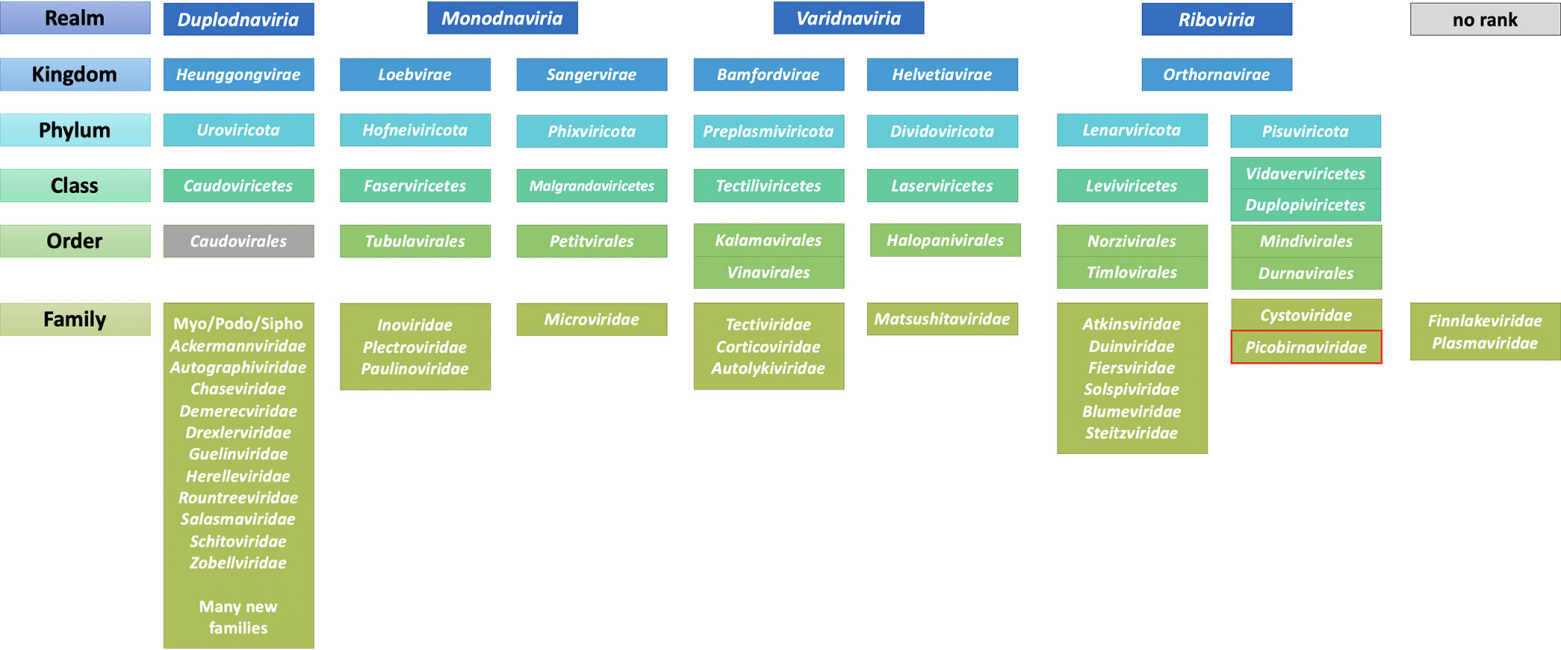
Overview of the virus ranks containing bacteriophages as of Master Species List 36 (https://talk.ictvonline.org/files/master-species-lists/m/msl/12314 [accessed June 2021]). The order *Caudovirales* is indicated in gray as it is scheduled for deletion. The family *Picobirnaviridae* outlined in red was originally recognized as a family of animal-associated viruses, but is now bioinformatically predicted to be made up of bacteriophages.

In 2020, a major step was taken in virus taxonomy with the implementation of higher ranks, the so-called megataxonomy of viruses (1, 2), providing 15 hierarchical ranks in which to classify all viruses. The known diversity of phages is now spread over four realms (*Duplodnaviria*, *Monodnaviria*, *Varidnaviria*, and *Riboviria*) that encompass six kingdoms and seven phyla (Fig. 1). The most commonly isolated phages, double-stranded DNA (dsDNA) tailed bacteriophages with a HK97-like major capsid protein, are unified in the class *Caudoviricetes*, at the time of writing equivalent with the order *Caudovirales*. At the family level, which is often used as a bin to visualize metagenomics data, the phage taxonomy is undergoing a rapid revolution from morphology-based classification in favor of a genome-based classification (3). As a result, many new families are being created so that members of the same family share a set of core genes, which is not the case with the classification into the families *Myoviridae*, *Podoviridae*, and *Siphoviridae*, which are scheduled to be abolished. At the ranks of species and genus, nucleotide identity-based demarcation criteria have been implemented that allow for systematic binning of metagenome data at these ranks (3–6). These levels are the most well-curated and comprehensive, reflected in the high number of proposals describing new genera in recent years (7–9). However, since phage (and all virus) taxonomy is done *post hoc*, i.e., new phage isolates are described and published first and only then classified by committee, the latest taxonomy database will always lag behind the “known” phage diversity.

As a result of the changes to and limitations of taxonomy, the current phage taxonomy database, as described on the website of the ICTV (International Committee on Taxonomy of Viruses) (ictvonline.org) and implemented by NCBI Taxonomy (10), is what I can only describe as a bit of a mixed bag. Given the large amounts of manual curation involved with classification and nomenclature, some parts of the phage sequence space have been tackled recently and are thus clearly defined, while others are not. This poses a lot of issues for the correct interpretation of microbiome/virome data, exactly because family-level descriptions are so often used (including in the past by myself). Unfortunately, some of these family-level analyses are wrong—for now—and should be avoided or at the very least manually curated, which I will explore in the example below.

## AN EXAMPLE OF A HEALTHY HUMAN GUT PHAGEOME: WHERE CAN THE ANALYSES GO WRONG?

**FIG 2:**
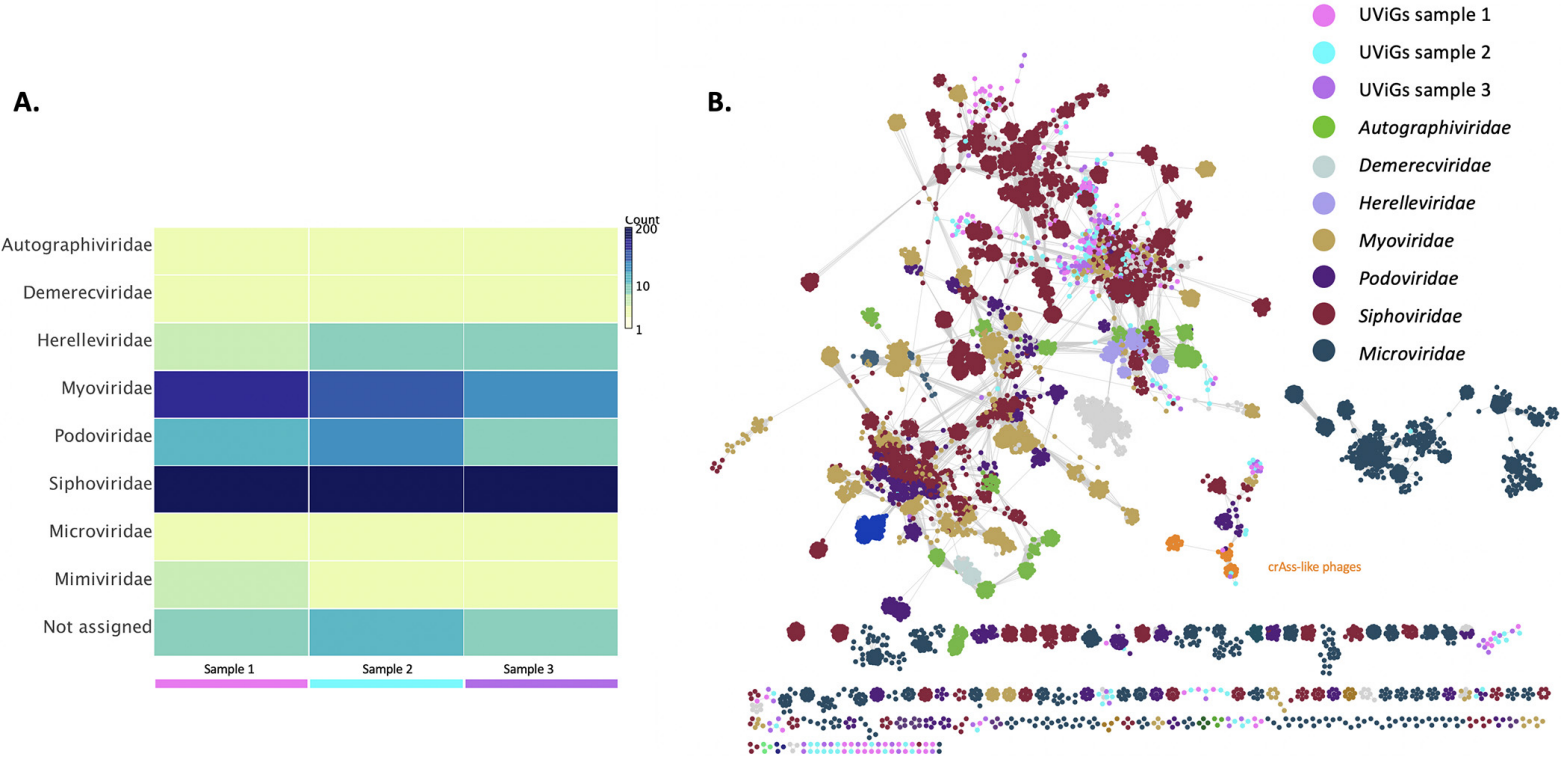
Phage diversity analysis of three gut phageome samples. (A) Heatmap of the number of uncultivated virus genomes (UViGs) per family grouped at the viral family level using Diamond BLASTX against the viral RefSeq database release 99 and lowest common ancestor assignment using Megan6. (B) vConTACT2 network analysis of UViGs from three samples and all published complete phage genomes of the INPHARED pipeline on 24 January 2021. Families are colored according to the INPHARED metadata, with selected families indicated in the legend. Archaeal viruses and the taxa of nontailed phages except for microviruses were removed.

In this example, I am using three distinct phage communities extracted from metagenome sequencing data sets from fecal samples from three healthy individuals (data derived from T. Brown and E.M. Adriaenssens, unpublished data). For each sample/individual, we assembled and validated the phage genomes using megahit and VirSorter, respectively (11, 12). Figure 2 shows two different analyses and visualizations of the same data: (i) heatmap of a reference-based assignment of contigs to a viral family using Diamond and Megan (13, 14), (ii) network representation of contigs and reference genomes as nodes (circles) connected by edges that represent shared protein clusters using vConTACT2 and the INPHARED pipeline (15, 16). What is immediately obvious from this comparison is that the dsDNA families *Myoviridae*, *Podoviridae*, and *Siphoviridae* and also the single-stranded DNA (ssDNA) family *Microviridae*, which are in a single bin in the heatmap (Fig. 2A) do not represent the phage sequence space well as they are separated across multiple clusters in the network (Fig. 2B). With the current taxonomic organization, two phages can belong to the same family and share no core proteins (or at least none that we can detect with sequence-based tools). The newer genome-based families are more cohesive across the network, but not the family *Autographiviridae*, which may get split further.

There are additional interesting observations that can be derived from this example. While the phage communities in the three healthy individuals are similar, they are not identical. There are also multiple clusters of related phages that bear no resemblance with database phages, which in the analysis in Fig. 2A are all classed together in the “Not assigned” bin, losing resolution. Where the two analyses are in agreement is the observation that siphoviruses dominate the gut phageome.

Given the realities of phage taxonomy and microbiome analyses, I can make the following recommendations.

- Do not rely on automated family-level binning approaches for analysis of the phageome
- Use different clustering methods for diversity analyses:
  - Nucleotide level clustering for species (95%) and genus (70%)
  - Shared predicted protein content for subfamilies and families
  - Deep rooted phylogenies of marker genes such as the terminase large subunit (*Caudoviricetes*) or capsid proteins for higher-order classifications
- Use multiple tools for exploration to reduce biases
- Take advantage of additional databases for higher resolution analyses (see examples below)

## GLOBAL HUMAN GUT PHAGE DIVERSITY AND crAssphages: ARE WE SPEAKING THE SAME LANGUAGE?

The recent surge in interest in bacteriophage research has led to the creation of a number of overlapping or competing databases describing the gut virome, which allow for additional resolution of gut phageome analyses. The Gut Virome Database (GVD) contains 33,242 viral populations from 1,986 individuals (17). The Cenote Human Virome Database (CHVD) comprises 45,033 viral operational taxonomic units (vOTUs) from all human body sites (18). The human Gut Phage Database is currently the largest gut-specific database with 142,809 nonredundant phage genomes assembled from 28,060 metagenomes and 2,898 bacterial genomes, of which 13,429 were classified as complete and a further 27,999 were classified as high quality by CheckV (19, 20). Another recent study assembled 3,738 complete phage genomes from 5,742 metagenomes (21). The most recent database is the Metagenomic Gut Virus (MGV) catalogue containing 189,680 (partial) genomes grouped into 54,118 species-level vOTUs (22). The MGV paper recognizes overlap and complementarity of the different databases and highlights the need for a unified and standardized resource, a sentiment I echo with enthusiasm.

In the papers describing these databases, often specific clades of phages are highlighted. For example, the GVD describes 70 crAssphage populations clustered into 12 viral clusters, but no single population shared across individuals (17). This is echoed by the analyses of the CHVD and GPD (18, 19), with the latter identifying a new clade dubbed Gubaphage that is distantly related to crAss-like phages. These descriptions across multiple publications and databases leave us in a Babel-like situation that, for instance, leaves us pondering what the term “crAssphage” actually means. When first described, it was posited as the most abundant human gut-associated phage (23, 24). However, the first cultured crAssphage, *Bacteriodes* phage phicrAss001 showed no nucleotide sequence similarity with the original crAssphage (25). Combining information from metagenomics studies and culturing approaches and driven by a collaboration across multiple research groups, the newly formed “Crassvirales Study Group” of the ICTV has submitted a proposal to create a new order, called *Crassvirales*, divided into multiple families, genera, and species (2021.022B.v1.Crassvirales, https://talk.ictvonline.org/files/proposals/taxonomy_proposals_prokaryote1/ [accessed June 2021]), allowing a taxonomic framework to facilitate the semantics associated with this group of phages (indicated in orange in Fig. 2B). It is my hope that this classification will normalize descriptions of crAss-like phages across publications and facilitate our understanding of this highly interesting group of phages.

## CONCLUSIONS AND PERSPECTIVES

In my—perhaps biased—opinion, both the phage community and the microbiome community need a well-curated genome-based taxonomic classification framework for phages. Put more strongly, taxonomy is the language that binds us together and will allow us to understand each other’s studies. In future, it is my hope that we can use the taxonomic framework to identify multiple sets of phages that are of importance to human health and disease, whether they are biomarkers for a healthy gut, indicative of a diseased state, or candidates for phage therapy. While this analysis was focused on the human gut, the taxonomic framework is not and will be essential in any environment.

I will leave the reader with three questions that we, as a community, need to answer so that we can understand each other across diverging fields of phage-related research:

- What is a phage?
- What is a viral family?
- When can we confidently say that a phage (or other type of virus) is present in a sample?
